# (Acetato-κ*O*)(2-{[2-(dimethyl­amino)­ethyl­imino](phen­yl)meth­yl}-5-methoxy­phenolato-κ^3^ *N*,*N*′,*O* ^1^)copper(II)

**DOI:** 10.1107/S1600536808033114

**Published:** 2008-10-18

**Authors:** Chieh-Shen Lin, Chia-Her Lin, Jui-Hsien Huang, Bao-Tsan Ko

**Affiliations:** a Department of Chemistry, National Changhua University of Education, Changhua 500, Taiwan; b Department of Chemistry, Chung-Yuan Christian University, Chung-Li 320, Taiwan

## Abstract

The Cu^II^ atom in the title complex, [Cu(C_18_H_21_N_2_O_2_)(C_2_H_3_O_2_)], is tetra­coordinated by two N atoms and two O atoms, of which one O atom is attributed to the acetate group and the other atoms are from the tridentate salicylideneiminate ligand, forming a slight distorted square-planar environment. The other acetate O atom exhibits a very weak intra­molecular inter­action toward the Cu atom, the Cu—O distance of 2.771 (2) Å being shorter than the van der Waals radii for Cu and O atoms (2.92 Å). Furthermore, there are weak inter­molecular inter­actions, in which the bonding O atom of the acetate group can bridge to the Cu atom of another complex, and the distance of 2.523 (2) Å is about 0.4 Å shorter than the van der Waals Cu—O distance in other crystal structures.

## Related literature

For general background, see: Coates & Moore (2004 ▶); Darensbourg *et al.* (2001 ▶); Inoue *et al.* (1969 ▶); Shen *et al.* (2003 ▶). For related structures, see: Chen *et al.* (2006 ▶); Luo *et al.* (1998 ▶, 1999 ▶).
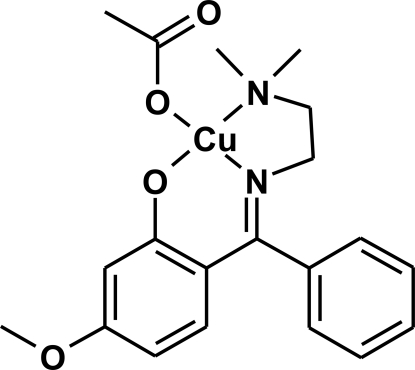


## Experimental

### 

#### Crystal data

[Cu(C_18_H_21_N_2_O_2_)(C_2_H_3_O_2_)]
*M* *_r_* = 419.96
Monoclinic, 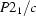

*a* = 11.9721 (16) Å
*b* = 15.674 (2) Å
*c* = 10.6346 (14) Å
β = 102.655 (3)°
*V* = 1947.1 (4) Å^3^
*Z* = 4
Mo *K*α radiation
μ = 1.15 mm^−1^
*T* = 293 (2) K
0.40 × 0.30 × 0.20 mm

#### Data collection

Bruker SMART 1000 CCD diffractometer
Absorption correction: multi-scan (*SADABS*; Sheldrick, 1996 ▶) *T*_min_ = 0.656, *T*_max_ = 0.803
11008 measured reflections
3822 independent reflections
2673 reflections with *I* > 2σ(*I*)
*R*_int_ = 0.049

#### Refinement

*R*[*F* ^2^ > 2σ(*F* ^2^)] = 0.040
*wR*(*F* ^2^) = 0.103
*S* = 1.02
3822 reflections
244 parameters
H-atom parameters constrained
Δρ_max_ = 0.47 e Å^−3^
Δρ_min_ = −0.40 e Å^−3^

### 

Data collection: *SMART* (Bruker, 1999 ▶); cell refinement: *SAINT* (Bruker, 1999 ▶); data reduction: *SAINT*; program(s) used to solve structure: *SHELXS97* (Sheldrick, 2008 ▶); program(s) used to refine structure: *SHELXL97* (Sheldrick, 2008 ▶); molecular graphics: *SHELXTL* (Sheldrick, 2008 ▶); software used to prepare material for publication: *SHELXTL*.

## Supplementary Material

Crystal structure: contains datablocks global, I. DOI: 10.1107/S1600536808033114/rk2108sup1.cif

Structure factors: contains datablocks I. DOI: 10.1107/S1600536808033114/rk2108Isup2.hkl

Additional supplementary materials:  crystallographic information; 3D view; checkCIF report

## Figures and Tables

**Fig. 1.**
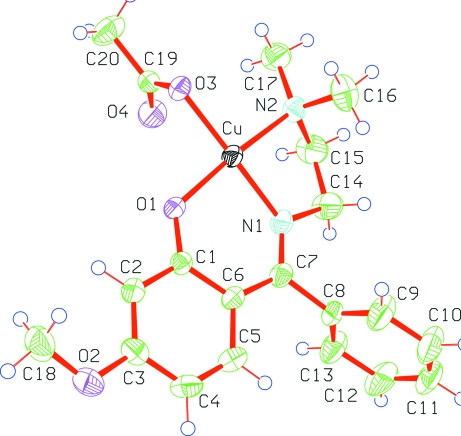
A view of the molecular structure of I with the atom numbering scheme. Displacement ellipsoids are drawn at the 50% probability level. H atoms are presented as a small spheres of arbitrary radius.

**Fig. 2.**
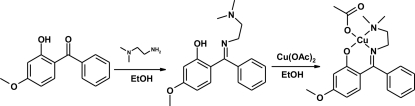
The synthetic route of the title Cu complex.

## supplementary crystallographic information

### Comment

Carbon dioxide is the most abundant carbon resource in the atmosphere and is used by green plants and anaerobic bacteria for chemical production on a massive scale. In contrast, industrial and laboratory utilization of CO_2_ as a chemical feedstock is rare. The reuse and recovery of CO_2_ have received much attention from the viewpoint of carbon resources and environmental problems during the last two decades of the twentieth century. In particular, the catalytic coupling of CO_2_ with heterocycles has been discovered and investigated over the past 35 years (Inoue *et al.*, 1969). One of the major successes is the utilization of epoxides and CO_2_ as starting materials to prepare the polycarbonates and/or cyclic carbonates in the presence of a transition metal catalyst. However, only a few metals, including Al, Cr, Co, Mg, Li, Zn, Cu, and Cd (Coates & Moore, 2004) are active for the coupling of epoxides and CO_2_. Recently, Darensbourg *et al.*, (2001) disclosed the synthesis, characterization and catalytic studies of a number of bis(salicylaldiminato)zinc complexes, in which the most active catalyst for co–polymerization of cyclohexene oxide and CO_2_ giving poly(cyclohexene carbonate) (>99% carbonate linkages, *Mn* = 41000 g^.^mol^-1^, *Mw*/*Mn* = 10.3) with a turnover frequency of 6.9 h^-1^. In addition, Shen *et al.* (2003) reported that binaphthyldiaminosalen–type Zn, Cu, and Co complexes efficiently catalyzed reactions of epoxides with CO_2_ to achieve five–membered ring cyclic carbonates in the presence of various catalytic amounts of organic bases. Most recently, Chen *et al.*, (2006) has synthesized a series of Schiff base zinc complexes which have shown high activity in the ring–opening poymerization of lactide (Chen *et al.*, 2006). We report herein the synthesis and crystal structure study of a *N,N,O*–tridentate Schiff base Cu^II^ complex (I), a potential catalyst for CO_2_/epoxide coupling copolymerization (Fig. 1).

The solid structure of I reveals a monomeric Cu^II^ complex (Fig. 1) containing a six–member and five–member ring coordinated from the tridentate salicylideneiminate ligand. The geometry around Cu atom is tetracoordinated with a slight distorted square planar environment in which two nitrogen atoms and two oxygen atoms are almost coplanar. The sums of bond angles around Cu center are 359.7 (1)°. The distances between the Cu atom and O1, O3, N1 and N2 are 1.908 (2), 1.968 (2), 2.073 (3), 1.969 (3)Å, respectively. These bond distances are similar to those found in other Schiff base Cu^II^ complexes (Luo *et al.*, 1998). The other acetate's oxygen, O4 shows very weak intramolecular contact with Cu (Cu···O4 = 2.771 (2)Å) in comparison with Van der Waals contact (2.92Å) for Cu···O. In addition, there are weak intermolecular interactions, in which the bonding oxygen (O3) of acetate group can be bridged to the Cu atom of another complex and the distance (2.523 (2) Å) is about 0.4 Å shorter than Van der Waals contact of Cu···O in the other crystal structure.

### Experimental

The ligand, 5–methoxy–2–{1–[2–(dimethylamino)ethylimino]benzyl}phenol was prepared by the reaction in which 2–dimethylaminoethylamine (1.95 g, 22.1 mmol) and 5–methoxy–2–hydroxybenzophenone (4.60 g, 20.2 mmol) in refluxed ethanol (30 ml) for 24 h (Fig. 2). Volatile materials were removed under vacuum and the resulting solid was recrystallized from slowly cooling a hot hexane (40 ml) solution giving yellow powders (yield: 71%). The title complex was synthesized by the following procedures: 5–methoxy–2–{1–[2–(dimethylamino)ethylimino]benzyl}phenol (0.597 g, 2.0 mmol) and Cu(O*Ac*)_2_^.^2H_2_O (0.398 g, 2.0 mmol) was refluxed in *Et*OH (30 ml) for 3 h and the volatile materials were removed under vacuum giving green crystalline solid (Fig. 2). The resulting precipitate was crystallized from *Et*OH to yield green crystals.

### Refinement

The C–bound H atoms were placed in calculated positions (C—H = 0.93-0.96 Å) and included in the refinement in the riding–model approximation, with *U*_iso_(H) = 1.2 or 1.5U_eq_(C).

### Crystal data

**Table:**

[Cu(C_18_H_21_N_2_O_2_)(C_2_H_3_O_2_)]	*F*(000) = 876
*M**_r_* = 419.96	*D*_x_ = 1.433 Mg m^−^^3^
Monoclinic, *P*2_1_/*c*	Mo *K*α radiation, λ = 0.71073 Å
Hall symbol: -P 2ybc	Cell parameters from 3118 reflections
*a* = 11.9721 (16) Å	θ = 2.2–25.4°
*b* = 15.674 (2) Å	µ = 1.15 mm^−^^1^
*c* = 10.6346 (14) Å	*T* = 293 K
β = 102.655 (3)°	Prism, green
*V* = 1947.1 (4) Å^3^	0.40 × 0.30 × 0.20 mm
*Z* = 4	

### Data collection

**Table:**

Bruker SMART 1000 CCD diffractometer	3822 independent reflections
Radiation source: Fine–focus sealed tube	2673 reflections with *I* > 2σ(*I*)
Graphite	*R*_int_ = 0.049
φ and ω scans	θ_max_ = 26.0°, θ_min_ = 2.2°
Absorption correction: multi-scan (SADABS; Sheldrick, 1996)	*h* = −14→14
*T*_min_ = 0.656, *T*_max_ = 0.803	*k* = −18→19
11008 measured reflections	*l* = −8→13

### Refinement

**Table:**

Refinement on *F*^2^	Primary atom site location: Direct
Least-squares matrix: Full	Secondary atom site location: Difmap
*R*[*F*^2^ > 2σ(*F*^2^)] = 0.040	Hydrogen site location: Geom
*wR*(*F*^2^) = 0.103	H-atom parameters constrained
*S* = 1.02	*w* = 1/[σ^2^(*F*_o_^2^) + (0.05*P*)^2^] where *P* = (*F*_o_^2^ + 2*F*_c_^2^)/3
3822 reflections	(Δ/σ)_max_ = 0.001
244 parameters	Δρ_max_ = 0.47 e Å^−^^3^
0 restraints	Δρ_min_ = −0.40 e Å^−^^3^

### Special details

**Table:**

Geometry. All s.u.'s (except the s.u. in the dihedral angle between two l.s. planes) are estimated using the full covariance matrix. The cell s.u.'s are taken into account individually in the estimation of s.u.'s in distances, angles and torsion angles; correlations between s.u.'s in cell parameters are only used when they are defined by crystal symmetry. An approximate (isotropic) treatment of cell s.u.'s is used for estimating s.u.'s involving l.s. planes.
Refinement. Refinement of *F*^2^ against ALL reflections. The weighted *R*–factor *wR* and goodness of fit *S* are based on *F*^2^, conventional *R*–factors *R* are based on *F*, with *F* set to zero for negative *F*^2^. The threshold expression of *F*^2^ > σ(*F*^2^) is used only for calculating *R*–factors(gt) *etc*. and is not relevant to the choice of reflections for refinement. *R*–factors based on *F*^2^ are statistically about twice as large as those based on *F*, and *R*–factors based on ALL data will be even larger.

### Fractional atomic coordinates and isotropic or equivalent isotropic displacement parameters (Å^2^)

**Table:**

	*x*	*y*	*z*	*U*_iso_*/*U*_eq_	
Cu	0.93747 (3)	0.10053 (2)	0.02371 (4)	0.02773 (13)	
O1	0.92068 (16)	0.06311 (14)	0.1893 (2)	0.0332 (5)	
O2	0.7404 (2)	−0.04350 (17)	0.5113 (2)	0.0474 (6)	
O3	1.09237 (16)	0.05131 (13)	0.0552 (2)	0.0303 (5)	
O4	1.1426 (2)	0.17945 (16)	0.1343 (2)	0.0467 (6)	
N1	0.7889 (2)	0.15948 (18)	−0.0052 (3)	0.0379 (7)	
N2	0.9457 (2)	0.15348 (17)	−0.1526 (3)	0.0333 (6)	
C1	0.8276 (2)	0.05790 (19)	0.2325 (3)	0.0268 (7)	
C2	0.8331 (3)	0.0119 (2)	0.3478 (3)	0.0327 (7)	
H2A	0.9021	−0.0132	0.3880	0.039*	
C3	0.7400 (3)	0.0029 (2)	0.4025 (3)	0.0355 (8)	
C4	0.6361 (3)	0.0424 (2)	0.3444 (3)	0.0434 (9)	
H4A	0.5733	0.0386	0.3825	0.052*	
C5	0.6277 (3)	0.0858 (2)	0.2337 (4)	0.0392 (8)	
H5A	0.5581	0.1112	0.1967	0.047*	
C6	0.7199 (2)	0.0949 (2)	0.1702 (3)	0.0290 (7)	
C7	0.7052 (3)	0.1444 (2)	0.0533 (3)	0.0321 (7)	
C8	0.5879 (2)	0.1779 (2)	−0.0065 (3)	0.0348 (8)	
C9	0.5162 (3)	0.1296 (3)	−0.0985 (4)	0.0603 (12)	
H9A	0.5396	0.0757	−0.1189	0.072*	
C10	0.4106 (3)	0.1598 (3)	−0.1607 (4)	0.0675 (13)	
H10A	0.3635	0.1267	−0.2232	0.081*	
C11	0.3748 (3)	0.2386 (3)	−0.1307 (4)	0.0581 (11)	
H11A	0.3038	0.2593	−0.1733	0.070*	
C12	0.4432 (3)	0.2866 (3)	−0.0384 (4)	0.0635 (12)	
H12A	0.4181	0.3397	−0.0169	0.076*	
C13	0.5505 (3)	0.2568 (3)	0.0241 (4)	0.0527 (10)	
H13A	0.5970	0.2902	0.0868	0.063*	
C14	0.7695 (3)	0.2182 (3)	−0.1169 (4)	0.0592 (12)	
H14A	0.7370	0.2715	−0.0952	0.071*	
H14B	0.7167	0.1929	−0.1896	0.071*	
C15	0.8852 (3)	0.2344 (2)	−0.1512 (4)	0.0535 (10)	
H15A	0.8737	0.2613	−0.2352	0.064*	
H15B	0.9306	0.2726	−0.0883	0.064*	
C16	0.8860 (3)	0.0981 (3)	−0.2608 (4)	0.0561 (11)	
H16A	0.8906	0.1237	−0.3415	0.084*	
H16B	0.8071	0.0918	−0.2567	0.084*	
H16C	0.9219	0.0430	−0.2539	0.084*	
C17	1.0606 (3)	0.1696 (3)	−0.1764 (4)	0.0522 (10)	
H17A	1.0535	0.1941	−0.2606	0.078*	
H17B	1.1020	0.1169	−0.1717	0.078*	
H17C	1.1011	0.2084	−0.1126	0.078*	
C18	0.8349 (3)	−0.1007 (3)	0.5543 (4)	0.0537 (10)	
H18A	0.8254	−0.1296	0.6309	0.081*	
H18B	0.9050	−0.0688	0.5726	0.081*	
H18C	0.8372	−0.1418	0.4881	0.081*	
C19	1.1673 (3)	0.1069 (2)	0.1073 (3)	0.0329 (7)	
C20	1.2906 (3)	0.0780 (3)	0.1325 (4)	0.0571 (12)	
H20A	1.3396	0.1237	0.1713	0.086*	
H20B	1.3096	0.0620	0.0525	0.086*	
H20C	1.3008	0.0298	0.1896	0.086*	

### Atomic displacement parameters (Å^2^)

**Table:**

	*U*^11^	*U*^22^	*U*^33^	*U*^12^	*U*^13^	*U*^23^
Cu	0.0244 (2)	0.0309 (2)	0.0274 (2)	0.00498 (16)	0.00454 (14)	0.00588 (17)
O1	0.0233 (11)	0.0480 (14)	0.0286 (13)	0.0067 (10)	0.0060 (9)	0.0079 (10)
O2	0.0479 (14)	0.0579 (18)	0.0401 (15)	−0.0047 (12)	0.0181 (11)	0.0103 (13)
O3	0.0234 (10)	0.0327 (13)	0.0332 (13)	−0.0008 (9)	0.0027 (9)	0.0013 (10)
O4	0.0470 (15)	0.0407 (16)	0.0502 (17)	0.0011 (12)	0.0061 (12)	−0.0130 (12)
N1	0.0352 (15)	0.0444 (19)	0.0333 (17)	0.0152 (13)	0.0055 (12)	0.0123 (13)
N2	0.0354 (15)	0.0289 (16)	0.0344 (17)	0.0033 (12)	0.0053 (12)	0.0072 (12)
C1	0.0257 (15)	0.0265 (17)	0.0271 (18)	−0.0019 (12)	0.0034 (13)	−0.0033 (13)
C2	0.0295 (16)	0.037 (2)	0.0309 (19)	0.0011 (14)	0.0042 (13)	0.0023 (14)
C3	0.0411 (19)	0.035 (2)	0.032 (2)	−0.0092 (15)	0.0106 (15)	−0.0030 (15)
C4	0.0347 (19)	0.050 (2)	0.052 (2)	0.0000 (16)	0.0217 (16)	0.0017 (19)
C5	0.0282 (17)	0.040 (2)	0.050 (2)	0.0031 (14)	0.0106 (15)	0.0002 (17)
C6	0.0242 (15)	0.0284 (18)	0.0334 (18)	0.0011 (13)	0.0039 (13)	−0.0032 (14)
C7	0.0293 (16)	0.0291 (19)	0.036 (2)	0.0068 (13)	0.0024 (14)	−0.0061 (15)
C8	0.0277 (17)	0.041 (2)	0.033 (2)	0.0092 (14)	0.0008 (14)	0.0031 (15)
C9	0.041 (2)	0.059 (3)	0.071 (3)	0.0148 (19)	−0.011 (2)	−0.026 (2)
C10	0.043 (2)	0.077 (3)	0.069 (3)	0.013 (2)	−0.017 (2)	−0.021 (2)
C11	0.0314 (19)	0.070 (3)	0.065 (3)	0.0144 (19)	−0.0067 (18)	0.011 (2)
C12	0.045 (2)	0.049 (3)	0.090 (3)	0.0256 (19)	0.002 (2)	−0.003 (2)
C13	0.038 (2)	0.047 (2)	0.065 (3)	0.0128 (17)	−0.0054 (18)	−0.011 (2)
C14	0.059 (2)	0.071 (3)	0.053 (3)	0.034 (2)	0.0219 (19)	0.034 (2)
C15	0.068 (3)	0.041 (2)	0.055 (3)	0.0165 (19)	0.022 (2)	0.0177 (19)
C16	0.070 (3)	0.053 (3)	0.041 (2)	−0.003 (2)	0.0019 (19)	0.0058 (19)
C17	0.048 (2)	0.062 (3)	0.051 (3)	0.0074 (19)	0.0203 (18)	0.021 (2)
C18	0.058 (2)	0.062 (3)	0.041 (2)	−0.005 (2)	0.0095 (18)	0.015 (2)
C19	0.0274 (16)	0.045 (2)	0.0265 (18)	−0.0012 (15)	0.0061 (13)	−0.0016 (16)
C20	0.0288 (19)	0.063 (3)	0.076 (3)	0.0009 (17)	0.0047 (18)	−0.011 (2)

### Geometric parameters (Å, °)

**Table:**

Cu—O1	1.908 (2)	C9—C10	1.377 (5)
Cu—N1	1.968 (3)	C9—H9A	0.9300
Cu—O3	1.968 (2)	C10—C11	1.368 (6)
Cu—N2	2.073 (3)	C10—H10A	0.9300
O1—C1	1.298 (3)	C11—C12	1.359 (5)
O2—C3	1.366 (4)	C11—H11A	0.9300
O2—C18	1.437 (4)	C12—C13	1.392 (5)
O3—C19	1.287 (4)	C12—H12A	0.9300
O4—C19	1.224 (4)	C13—H13A	0.9300
N1—C7	1.312 (4)	C14—C15	1.529 (5)
N1—C14	1.481 (4)	C14—H14A	0.9700
N2—C15	1.463 (4)	C14—H14B	0.9700
N2—C17	1.475 (4)	C15—H15A	0.9700
N2—C16	1.493 (4)	C15—H15B	0.9700
C1—C2	1.412 (4)	C16—H16A	0.9600
C1—C6	1.436 (4)	C16—H16B	0.9600
C2—C3	1.374 (4)	C16—H16C	0.9600
C2—H2A	0.9300	C17—H17A	0.9600
C3—C4	1.405 (5)	C17—H17B	0.9600
C4—C5	1.345 (5)	C17—H17C	0.9600
C4—H4A	0.9300	C18—H18A	0.9600
C5—C6	1.421 (4)	C18—H18B	0.9600
C5—H5A	0.9300	C18—H18C	0.9600
C6—C7	1.443 (4)	C19—C20	1.510 (4)
C7—C8	1.505 (4)	C20—H20A	0.9600
C8—C13	1.378 (5)	C20—H20B	0.9600
C8—C9	1.378 (5)	C20—H20C	0.9600
			
O1—Cu—N1	90.82 (10)	C10—C11—C12	119.9 (3)
O1—Cu—O3	90.49 (8)	C10—C11—H11A	120.0
N1—Cu—O3	175.05 (11)	C12—C11—H11A	120.0
O1—Cu—N2	173.51 (10)	C13—C12—C11	120.4 (4)
N1—Cu—N2	83.74 (11)	C13—C12—H12A	119.8
O3—Cu—N2	94.64 (9)	C11—C12—H12A	119.8
C1—O1—Cu	128.17 (19)	C8—C13—C12	120.1 (3)
C3—O2—C18	117.2 (3)	C8—C13—H13A	119.9
C19—O3—Cu	110.5 (2)	C12—C13—H13A	119.9
C7—N1—C14	119.4 (3)	N1—C14—C15	107.7 (3)
C7—N1—Cu	126.8 (2)	N1—C14—H14A	110.2
C14—N1—Cu	113.3 (2)	C15—C14—H14A	110.2
C15—N2—C17	109.6 (3)	N1—C14—H14B	110.2
C15—N2—C16	111.0 (3)	C15—C14—H14B	110.2
C17—N2—C16	105.9 (3)	H14A—C14—H14B	108.5
C15—N2—Cu	102.5 (2)	N2—C15—C14	109.6 (3)
C17—N2—Cu	117.1 (2)	N2—C15—H15A	109.8
C16—N2—Cu	110.8 (2)	C14—C15—H15A	109.8
O1—C1—C2	117.3 (3)	N2—C15—H15B	109.8
O1—C1—C6	124.4 (3)	C14—C15—H15B	109.8
C2—C1—C6	118.3 (3)	H15A—C15—H15B	108.2
C3—C2—C1	122.2 (3)	N2—C16—H16A	109.5
C3—C2—H2A	118.9	N2—C16—H16B	109.5
C1—C2—H2A	118.9	H16A—C16—H16B	109.5
O2—C3—C2	124.1 (3)	N2—C16—H16C	109.5
O2—C3—C4	116.5 (3)	H16A—C16—H16C	109.5
C2—C3—C4	119.4 (3)	H16B—C16—H16C	109.5
C5—C4—C3	119.8 (3)	N2—C17—H17A	109.5
C5—C4—H4A	120.1	N2—C17—H17B	109.5
C3—C4—H4A	120.1	H17A—C17—H17B	109.5
C4—C5—C6	123.4 (3)	N2—C17—H17C	109.5
C4—C5—H5A	118.3	H17A—C17—H17C	109.5
C6—C5—H5A	118.3	H17B—C17—H17C	109.5
C7—C6—C5	120.2 (3)	O2—C18—H18A	109.5
C7—C6—C1	122.8 (3)	O2—C18—H18B	109.5
C5—C6—C1	116.9 (3)	H18A—C18—H18B	109.5
N1—C7—C6	123.0 (3)	O2—C18—H18C	109.5
N1—C7—C8	118.4 (3)	H18A—C18—H18C	109.5
C6—C7—C8	118.6 (3)	H18B—C18—H18C	109.5
C13—C8—C9	118.5 (3)	O4—C19—O3	123.3 (3)
C13—C8—C7	122.3 (3)	O4—C19—C20	120.9 (3)
C9—C8—C7	119.1 (3)	O3—C19—C20	115.8 (3)
C10—C9—C8	121.0 (4)	C19—C20—H20A	109.5
C10—C9—H9A	119.5	C19—C20—H20B	109.5
C8—C9—H9A	119.5	H20A—C20—H20B	109.5
C11—C10—C9	120.0 (4)	C19—C20—H20C	109.5
C11—C10—H10A	120.0	H20A—C20—H20C	109.5
C9—C10—H10A	120.0	H20B—C20—H20C	109.5
